# Comparison of Simulated Outcomes of Colorectal Cancer Surgery at the Highest-Performing vs Chosen Local Hospitals

**DOI:** 10.1001/jamanetworkopen.2022.55999

**Published:** 2023-02-15

**Authors:** Caitlin B. Finn, Chris Wirtalla, Sanford E. Roberts, Karole Collier, Shivan J. Mehta, Carmen E. Guerra, Edoardo Airoldi, Xu Zhang, Luke Keele, Cary B. Aarons, Shane T. Jensen, Rachel R. Kelz

**Affiliations:** 1 Center for Surgery and Health Economics, Department of Surgery, University of Pennsylvania, Philadelphia; 2 Leonard David Institute of Health Economics, University of Pennsylvania, Philadelphia; 3 Department of Medicine, University of Pennsylvania, Philadelphia; 4 Department of Statistical Science, Fox School of Business, Temple University, Philadelphia, Pennsylvania; 5 Department of Statistics and Data Science, The Wharton School at the University of Pennsylvania, Philadelphia

## Abstract

**Importance:**

Variation in outcomes across hospitals adversely affects surgical patients. The use of high-quality hospitals varies by population, which may contribute to surgical disparities.

**Objective:**

To simulate the implications of data-driven hospital selection for social welfare among patients who underwent colorectal cancer surgery.

**Design, Setting, and Participants:**

This economic evaluation used the hospital inpatient file from the Florida Agency for Health Care Administration. Surgical outcomes of patients who were treated between January 1, 2016, and December 31, 2018 (training cohort), were used to estimate hospital performance. Costs and benefits of care at alternative hospitals were assessed in patients who were treated between January 1, 2019, and December 31, 2019 (testing cohort). The cohorts comprised patients 18 years or older who underwent elective colorectal resection for benign or malignant neoplasms. Data were analyzed from March to October 2022.

**Exposures:**

Using hierarchical logistic regression, we estimated the implications of hospital selection for in-hospital mortality risk in patients in the training cohort. These estimates were applied to patients in the testing cohort using bayesian simulations to compare outcomes at each patient’s highest-performing and chosen local hospitals. Analyses were stratified by race and ethnicity to evaluate the potential implications for equity.

**Main Outcomes and Measures:**

The primary outcome was the mean patient-level change in social welfare, a composite measure balancing the value of reduced mortality with associated costs of care at higher-performing hospitals.

**Results:**

A total of 21 098 patients (mean [SD] age, 67.3 [12.0] years; 10 782 males [51.1%]; 2232 Black [10.6%] and 18 866 White [89.4%] individuals) who were treated at 178 hospitals were included. A higher-quality local hospital was identified for 3057 of 5000 patients (61.1%) in the testing cohort. Selecting the highest-performing hospital was associated with a 26.5% (95% CI, 24.5%-29.0%) relative reduction and 0.24% (95% CI, 0.23%-0.25%) absolute reduction in mortality risk. A mean amount of $1953 (95% CI, $1744-$2162) was gained in social welfare per patient treated. Simulated reassignment to a higher-quality local hospital was associated with a 23.5% (95% CI, 19.3%-32.9%) relative reduction and 0.26% (95% CI, 0.21%-0.30%) absolute reduction in mortality risk for Black patients, with $2427 (95% CI, $1697-$3158) gained in social welfare.

**Conclusions and Relevance:**

In this economic evaluation, using procedure-specific hospital performance as the primary factor in the selection of a local hospital for colorectal cancer surgery was associated with improved outcomes for both patients and society. Surgical outcomes data can be used to transform care and guide policy in colorectal cancer.

## Key Points

**Question:**

What are the implications of hospital selection for surgical outcomes in patients with colorectal cancer?

**Findings:**

In this economic evaluation involving 21 098 patients who underwent elective colorectal resection, simulated referral of these patients to higher-performing local hospitals for surgery was associated with improved welfare for patients and society.

**Meaning:**

Findings of this study suggest that procedure-specific outcomes data can be used to transform care and guide policy in colorectal cancer surgery.

## Teaser

This economic evaluation assesses the association of hospital referral choices with mortality risk, and the cost of these choices, in adults who underwent colorectal resection.

## Introduction

A patient’s chosen hospital for colorectal surgery affects their outcomes.^1,2,3,4,5^ For colectomy, in-hospital mortality ranges from 0.6% to 14.7% across hospitals,^6^ and mean 30-day costs vary 3-fold from $14 992 to $43 711.^6^ Hospital-level variation also affects risks of postoperative complications,^1,3^ readmission,^6^ and discharge to a skilled rehabilitation facility.^2^ Compared with White patients, Black patients are more likely to use lower-quality hospitals for surgery, even when a higher-quality hospital can be found closer to home.^7,8^ Interventions to promote selection of higher-quality hospitals may play a role in improved outcomes and reduced disparities.

Patients consider both surgeon and hospital factors when deciding where to receive care,^9^ but most prefer the hospital with superior outcomes.^10^ Up to 70% of patients rely on the expertise of their referring physician to direct them to high-performing surgeons.^11,12^ However, referring physicians rarely use volume or outcomes data to guide their recommendation, instead relying on subjective evaluations of clinical skill, reputation, and convenience.^13^ Furthermore, patient factors, such as advanced age, lack of transportation, and financial concerns, may be barriers to traveling to high-volume medical centers.^10,14^

The complex decision-making required for hospital selection has implications for society. Regionalization of complex surgery to a few specialized, high-volume medical centers has been proposed to improve outcomes, reduce disparities, and minimize societal costs of poor-quality care.^15,16,17^ However, traveling to high-volume centers is often costly for patients,^18,19^ making such care inaccessible for many.^10^ Alternatively, as proposed by Chi et al,^20^ a patient-centered referral system built on preferences for balancing proximity and outcomes may benefit patients and society alike.

For this study, we adopted a welfarist approach to evaluate the economic implications of using procedure-specific outcomes to guide hospital selection for surgery.^21^ We applied a societal perspective to simulate the implications of data-driven hospital selection for resource use among patients who underwent colorectal cancer surgery, balancing financial costs with associated survival benefits. By calculating the mean individual value gained through alternative hospital choices, we estimated the mean welfare benefit to society if each patient were treated at their highest-performing local hospital.

## Methods

### Sample and Setting

We included adult patients (≥18 years) who underwent elective resection for a colorectal neoplasm in Florida between January 1, 2016, and December 31, 2019, using the hospital inpatient file from the Florida Agency for Health Care Administration.^22^ This population-based economic evaluation was deemed exempt by the institutional review board at the University of Pennsylvania, and the informed consent requirement was waived because only deidentified data in the limited data set were used. We followed the Consolidated Health Economic Evaluation Reporting Standards (CHEERS) reporting guideline.^23^

Patients with *International Statistical Classification of Diseases and Related Health Problems, Tenth Revision (ICD-10)* codes for colonic resection^24^ were identified (eTable 1 in Supplement 1). Patients without a principal *ICD-10* diagnosis code for colorectal neoplasm were excluded (eTable 2 in Supplement 1). We included patients with either a benign or malignant neoplasm to capture both known and suspected malignant neoplasms given that pathologic diagnosis may be unknown during the surgical admission. We excluded patients presenting emergently to focus on those with time to consider their choice of hospital. We limited the cohort to Black and White patients to focus on established surgical disparities among Black patients.^25,26,27^

Patients were divided into 2 cohorts according to treatment year to model future hospital performance based on previous risk-adjusted outcomes. Prior hospital performance was assessed using a training cohort treated from January 1, 2016, to December 31, 2018. The estimated costs and benefits of care at alternative hospitals were assessed using a testing cohort treated in 2019.

We obtained patient demographic data, including age, self-reported race and ethnicity, sex, and insurance type, from the hospital inpatient file. Tumor type, location, and metastatic disease were identified using *International Statistical Classification of Diseases, Tenth Revision, Clinical Modification (ICD-10-CM)* diagnosis codes.^28^ Comorbidities present at admission were identified using the Elixhauser Comorbidity Index (range: 0 to ≥3, with the highest value indicating greatest number of comorbidities).^29^ Physical frailty was estimated using a modified claims-based frailty index.^30^ Descriptive hospital information was obtained from the American Hospital Association annual survey database.^31^

### Outcomes

The primary outcome was the mean patient-level change in social welfare achieved by simulated data-driven hospital selection, as described by Ho et al.^16^ Social welfare provides an overall economic assessment by balancing the simulated mortality benefit associated with data-driven hospital selection with costs of care at alternative hospitals. The measure of benefit was reduction in mortality during the index admission defined by the discharge status of *expired* (deceased). To quantify the financial gains associated with reduced in-hospital mortality, we estimated the life-years gained by multiplying predicted in-hospital survival (1 – predicted mortality) by the estimated postdischarge life expectancy. Life expectancy data were obtained from actuarial tables for patients who were matched by age and sex and multiplied by the expected 5-year survival for patients with locoregional (81.5%, a mean of 91.0% for local and 72.0% for regional) or metastatic (14.0%) colorectal cancer to adjust for cancer severity.^32^

We performed sensitivity analyses in which patients with benign neoplasms were assigned life expectancies without adjustment for cancer severity. The primary analysis valued each life-year at $100 000, and the sensitivity analyses used $50 000 and $150 000.^33^

The measures of harm included simulated patient-level differences in costs and travel. Costs were calculated by multiplying the total gross charges reported for each patient by the hospital’s operating cost-to-charge ratio (2016-2019 Centers for Medicare and Medicaid Services Final Rule Impact File)^34^ and then dividing by each hospital’s wage index for geographic normalization. Costs were inflated to 2019 dollars using the medical care component of the Consumer Price Index.^35^ We calculated the geographic distance in miles between the patient home and hospital zip codes.^36^ To focus on the quality of inpatient care, we simulated outcomes over the time of a patient’s hospitalization without discounting.

### Statistical Analysis

We performed bayesian simulation analyses to predict the risk of in-hospital mortality for each patient at their chosen or highest-performing local hospital. We constrained all models to consider hospitals located within 30 miles of the patient’s home to reflect patient preferences for local care. Given the instability of outcome estimation at low-volume hospitals, we required accepting hospitals to perform at least 30 colorectal resections (with an annual mean of 10 cases) in the training cohort. For the simulations, we made several assumptions: (1) the set of patients will remain fixed because referring physicians’ practices will be insensitive to a hospital location change, (2) patients will accept treatment at a better-quality hospital, and (3) surgery is the optimal treatment for patients with colorectal cancer who are seeking a cure.^37^

Each patient’s surgical outcome reflects both individual risk factors and added risks associated with care at their chosen hospital (eFigure in Supplement 1). To isolate the hospital’s association with risk, we used a hierarchical generalized logistic regression model with random effects (REs) at the hospital level to predict in-hospital mortality risk for patients in the training cohort (treated between January 1, 2016, and December 31, 2018). The model was adjusted for age, sex, race and ethnicity, insurance type, Elixhauser Comorbidity Index,^38^ frailty, operative approach (laparoscopic vs open), tumor type, procedure type, and presence of metastatic disease, which are collectively associated with a patient’s intrinsic risk of in-hospital mortality (ie, fixed effect [FE]). The model identified the implications of the hospital for outcomes through its RE hospital coefficients, which were assumed to follow a normal distribution with a mean and SD that were unique to that hospital. To describe hospital characteristics, hospitals were divided into quartiles by RE mean, such that quartile 1 hospitals were the highest performing and quartile 4 hospitals were the lowest performing.

The RE distribution based on hospital performance from 2016 to 2018 (ie, prior performance distribution) was used to predict the hospital’s performance in 2019. Due to the inherent uncertainty in predicting outcomes, we sampled from each hospital’s RE distribution 1000 times to obtain a posterior distribution of RE estimates for each possible hospital-patient combination. We then added the patient’s FE terms and converted the output to a probability (*e^RE^* ^+^ *^FE^* / 1 + *e^RE^* ^+^ *^FE^*) to yield the posterior predictive probability distribution of in-hospital mortality for each patient at their chosen and alternative hospitals. Next, we performed a realization of each patient’s predicted outcome by performing a Bernoulli trial (eg, coin flip) to obtain binary outcomes with a probability equal to each posterior predictive probability. Calculating the mean binary outcome over 1000 samples yielded a posterior predictive risk of in-hospital mortality for each patient at each local hospital.

We then compared the predicted in-hospital mortality risk at the chosen hospital vs alternative hospitals for each patient. We considered the hospital with the lowest predicted in-hospital mortality risk to be the highest-performing local hospital. By subtracting the predicted mortality risk at the highest-performing hospital from that at the chosen hospital, we obtained the potential mortality gains from optimized hospital selection for each patient.

To evaluate the difference in estimated costs between the chosen and highest-performing local hospital, we performed a similar sequence of analyses. We used hierarchical linear regression with hospital-level REs to estimate costs. Applying the mean hospital-level REs from the training cohort, we estimated the hospital contribution to cost. Combining these REs with the patient’s FEs for costs, we estimated each patient’s costs at their chosen and highest-performing local hospitals. To evaluate the distribution of welfare benefit across the population, we performed stratified analyses comparing Black with White patients.

We performed 1-way analyses of variance to describe continuous variables and χ^2^ tests to describe categorical variables. Hypothesis tests were 2-sided, with α = .05 indicating statistical significance. Data analyses were performed using Stata, version 17.0 (StataCorp LLC) and R, version 4.2.0 (R Core Team)^39^ from March to October 2022.

## Results

We included 21 098 patients (mean [SD] age, 67.3 [12.0] years), of whom 10 316 were female (48.9%) and 10 782 were male (51.1%). These patients self-identified as Black (2232 [10.6%]) or White (18 866 [89.4%]) individuals. Of these patients, 16 098 (76.3%) composed the training cohort and 5000 (23.7%) composed the testing cohort. The training and testing cohorts were similar in demographic characteristics, comorbidities, and illness severity (Table 1). Patients in the training cohort were slightly younger than those in the testing cohort (mean age [SD], 67.1 [12.0] years vs 67.8 [11.8] years; *P* = .001). Black patients were younger than White patients (mean [SD] age, 63.8 [11.7] years vs 67.7 [11.9] years; *P* < .001) but had more comorbidities (eTable 3 in Supplement 1).

**Table 1.** Characteristics of Patients in the Training and Testing Cohorts

Characteristic	No. (%)			*P* value
	Training cohort (2016-2018)	Testing cohort (2019)	All patients	
No. (%)	16 098 (76.3)	5000 (23.7)	21 098 (100)	
Age, mean (SD), y	67.1 (12.0)	67.8 (11.8)	67.3 (12.0)	.001
Sex				
Male	8226 (51.1)	2556 (51.1)	10 782 (51.1)	.98
Female	7872 (48.9)	2444 (48.9)	10 316 (48.9)	
Race and ethnicity				
Black	1719 (10.7)	513 (10.3)	2232 (10.6)	.40
White	14 379 (89.3)	4487 (89.7)	18 866 (89.4)	
Insurance type				
Medicare	9919 (61.6)	3235 (64.7)	13 154 (62.3)	.002
Medicaid	544 (3.4)	142 (2.8)	686 (3.3)	
Commercial	5113 (31.8)	1468 (29.4)	6581 (31.2)	
Self-pay	136 (0.8)	37 (0.7)	173 (0.8)	
Other^a^	386 (2.4)	118 (2.4)	504 (2.4)	
Elixhauser Comorbidity Index^b^				
0	857 (5.3)	246 (4.9)	1103 (5.2)	.18
1	2707 (16.8)	806 (16.1)	3513 (16.7)	
2	3605 (22.4)	1089 (21.8)	4694 (22.2)	
≥3	8929 (55.5)	2859 (57.2)	11 788 (55.9)	
Frailty	309 (1.9)	84 (1.7)	393 (1.9)	.27
Tumor type				
Malignant neoplasm of colon	8494 (52.8)	2747 (54.9)	11 241 (53.3)	.04
Malignant neoplasm of rectosigmoid junction	1088 (6.8)	325 (6.5)	1413 (6.7)	
Malignant neoplasm of rectum	1850 (11.5)	521 (10.4)	2371 (11.2)	
Benign or in situ neoplasm	4666 (29.0)	1407 (28.1)	6073 (28.8)	
Metastatic cancer	2277 (14.1)	681 (13.6)	2958 (14.0)	.35
Procedure type				
Colectomy	14 778 (91.8)	4621 (92.4)	19 399 (91.9)	.02
Other large bowel^c^	51 (0.3)	21 (0.4)	72 (0.3)	
Proctectomy	1218 (7.6)	353 (7.1)	1571 (7.4)	
Other small bowel^c^	51 (0.3)	<11 (<0.1)^d^	<62 (<0.1)	
Laparoscopic resection	8623 (53.6)	2615 (52.3)	11 238 (53.3)	.12

^a^ Other insurance type included nonpayment; Worker’s Compensation; Veterans Affairs; and other federal, state, or local government coverage.

^b^ Index range: 0 to ≥3, with the highest value indicating greatest number of comorbidities.

^c^ Other small bowel and other large bowel included anastomoses and repair of the small bowel and large bowel. A complete list of procedure codes defining these groups is provided in eTable 1 in Supplement 1.

^d^ Counts were greater than 0 but fewer than 11.

### Hospital Quality Assessment

Patients were treated at 178 hospitals, 177 (99.4%) of which were in urban locations (Table 2). Most hospitals were medium (81 [45.5%]) or large (74 [41.6%]) in bed size. Dividing hospitals into quartiles by their RE mean (SD) revealed that the unadjusted mortality risk ranged from 0.2% (0.4%) in quartile 1 hospitals to 2.8% (2.3%) in quartile 4 hospitals (*P* < .001). Hospitals in quartile 1 treated a higher median (interquartile interval [IQI]) number of patients over the study period than hospitals in quartile 4 (105 [66.5-156.5] vs 78 [35.8-166.3]; *P* < .001).

**Table 2.** Characteristics of Hospitals by In-Hospital Mortality Risk Stratified by RE Quartile^a^

Characteristic	RE, No. (%)					*P* value
	Quartile 1	Quartile 2	Quartile 3	Quartile 4	Total	
No. (%)	45 (25.3)	44 (24.7)	45 (25.3)	44 (24.7)	178 (100)	NA
Location type						
Urban	45 (100)	44 (100)	45 (100)	43 (97.7)	177 (99.4)	.38
Rural	0	0	0	<11 (<25.0)^b^	<11 (<6.2)^b^	
ACS-approved cancer center	26 (57.8)	15 (34.1)	17 (37.8)	23 (52.3)	81 (45.5)	.07
Cancer center affiliate	18 (40.0)	26 (59.1)	23 (51.1)	17 (38.6)	84 (47.2)	.17
Hospital size						
Small: <100 beds	<11 (<25.0)^b^	11 (25.0)	<11 (<25.0)^b^	<11 (<25.0)^b^	<44 (<24.7)	.007
Medium: 100-299 beds	25 (55.6)	20 (45.5)	22 (48.9)	14 (31.8)	81 (45.5)	
Large: ≥300 beds	19 (42.2)	13 (29.5)	16 (35.6)	26 (59.1)	74 (41.6)	
No. of patients treated, median (IQI)	105 (66.5-156.5)	36 (23.0-51.8)	18 (4.5-105.0)	78 (35.8-166.3)	59 (25.8-120.0)	<.001
% Mortality, mean (SD)	0.2 (0.4)	0.1 (0.3)	0.5 (0.7)	2.8 (2.3)	0.9 (0.2)	<.001

Abbreviations: ACS, American College of Surgeons; IQI, interquartile interval; NA, not applicable; RE, random effect.

^a^ Quartile 1 hospitals were the highest performing, and quartile 4 hospitals were the lowest performing.

^b^ Counts were greater than 0 but fewer than 11.

### Simulated Data-Driven Hospital Selection

We identified an alternative higher-performing local hospital for 3057 of 5000 patients (61.1% in the testing cohort (Table 3). Compared with those who remained at their chosen local hospital, patients who moved to a higher-performing local hospital were more likely to be male (54.3% vs 46.1%; *P* < .001), older (69.5 [11.4] years vs 65.0 (12.0) years; *P* < .001), and Medicare beneficiaries (71.4% vs 54.1%; *P* < .001). Patients who were eligible for care at a higher-performing hospital were less likely to have undergone a laparoscopic resection (47.6% vs 59.8%; *P* < .001). These 2 patient groups were similar in race and ethnicity, frailty, and disease severity.

**Table 3.** Characteristics of Patients in the Testing Cohort Who Were Eligible for Simulated Care at an Alternative Hospital

Characteristic	No. (%)		*P* value
	Remained at chosen hospital	Moved to alternative (higher-performing) hospital	
No. (%)	1943 (38.9)	3057 (61.1)	
Age, mean (SD), y	65.0 (12.0)	69.5 (11.4)	<.001
Sex			
Male	895 (46.1)	1661 (54.3)	<.001
Female	1048 (53.9)	1396 (45.7)	
Race and ethnicity			
Black	204 (10.5)	309 (10.1)	.66
White	1739 (89.5)	2748 (89.9)	
Insurance type			
Medicare	1051 (54.1)	2184 (71.4)	<.001
Medicaid	46 (2.4)	96 (3.1)	
Commercial	785 (40.4)	683 (22.3)	
Self-pay	19 (1.0)	18 (0.6)	
Other^a^	42 (2.2)	76 (2.5)	
Elixhauser Comorbidity Index^b^			
0	127 (6.5)	119 (3.9)	<.001
1	380 (19.6)	426 (13.9)	
2	477 (24.5)	612 (20.0)	
≥3	959 (49.4)	1900 (62.2)	
Frailty	26 (1.3)	58 (1.9)	.13
Tumor type			
Malignant neoplasm of colon	961 (49.5)	1786 (58.4)	<.001
Malignant neoplasm of rectosigmoid junction	173 (8.9)	152 (5.0)	
Malignant neoplasm of rectum	212 (10.9)	309 (10.1)	
Benign or in situ neoplasm	597 (30.7)	810 (26.5)	
Metastatic cancer	265 (13.6)	416 (13.6)	.98
Procedure type			
Colectomy	1789 (92.1)	2832 (92.6)	<.001
Other large bowel^c^	21 (1.1)	0	
Proctectomy	133 (6.8)	220 (7.2)	
Other small bowel^c^	0	<11 (<0.1)^d^	
Laparoscopic resection	1161 (59.8)	1454 (47.6)	<.001

^a^ Other insurance type included nonpayment; Worker’s Compensation; Veterans Affairs; and other federal, state, or local government coverage.

^b^ Index range: 0 to ≥3, with the highest value indicating greatest number of comorbidities.

^c^ Other small bowel and other large bowel included anastomoses and repair of the small bowel and large bowel. A complete list of procedure codes defining these groups is provided in eTable 1 in Supplement 1.

^d^ Counts were greater than 0 but fewer than 11.

At the hospital level, 106 hospitals (59.6%) experienced a net volume increase, while 72 (40.4%) either lost or had no change in volume (eTable 4 in Supplement 1). Hospitals that gained and lost patients in the simulation were similar in urbanicity. We observed patients moving from large hospitals to smaller hospitals and cancer center affiliates. Hospitals that gained patients saw an additional median (IQI) volume of 13 (7.0-19.3) patients, while the remaining hospitals lost a median (IQI) volume of 13 (27.0-3.30) patients over the 1-year simulation.

At the patient level, social welfare increased by a mean amount of $1953 (95% CI, $1744-$2162) per patient at the simulated highest-performing local hospitals (Table 4). The social welfare changes were associated with decreased in-hospital mortality risk from 0.91% (95% CI, 0.80%-1.01%) at the chosen hospital to 0.67% (95% CI, 0.57%-0.76%) at the simulated highest-performing hospital, a relative reduction of 26.5% (95% CI, 24.5%-29.0%) and absolute reduction of 0.24% (95% CI, 0.23%-0.25%). Estimated costs of care increased by $482 (95% CI, $325-$638). However, this increase was offset by the value of additional life gained due to avoidance of in-hospital mortality. Social welfare gains persisted in sensitivity analyses, which used alternative valuations of 1 life-year and estimations of life expectancy (eTables 5 and 6 in Supplement 1). At baseline, patients traveled a median (IQI) of 8.9 (5.0-15.7) miles to reach their chosen hospital. Selecting the highest-performing hospital within 30 miles was associated with increased travel of a median (IQI) distance of 10.8 (5.5-19.2) miles (*P* < .001), a difference of 1.9 (0.5-3.5) miles; to convert miles to kilometers, multiply by 1.6.

**Table 4.** Simulated Change in Patient-Level Outcomes After Data-Driven Hospital Selection

Outcome	Mean estimate (95% CI)		
	Chosen hospital	Highest-performing hospital	Difference
**Total population (N = 5000)**			
Mortality risk, %	0.91 (0.80 to 1.01)	0.67 (0.57 to 0.76)	−0.24 (−0.23 to −0.25)
Life expectancy, y	12.56 (12.34 to 12.77)	12.58 (12.37 to 12.80)	0.02 (0.02 to 0.03)
Costs, $	20 791 (20 536 to 21 046)	21 273 (21 027 to 21 519)	482 (325 to 638)
Change in social welfare, $^a^	NA	NA	1953 (1744 to 2162)
**Black population (n = 513)**			
Mortality risk, %	1.10 (0.68 to 1.53)	0.84 (0.45 to 1.23)	−0.26 (−0.21 to −0.30)
Life expectancy, y	13.65 (12.96 to 14.34)	13.68 (12.99 to 14.37)	0.03 (0.02 to 0.03)
Costs, $	22 688 (21 859 to 23 518)	23 038 (22 228 to 23 847)	349 (−183 to 882)
Change in social welfare, $^a^	NA	NA	2427 (1697 to 3158)
**White population (n = 4487)**			
Mortality risk, %	0.88 (0.78 to 0.99)	0.65 (0.55 to 0.74)	−0.24 (−0.25 to −0.22)
Life expectancy, y	12.43 (12.21 to 12.66)	12.46 (12.23 to 12.68)	0.02 (0.22 to 0.03)
Costs, $	20 574 (20 307 to 20 841)	21 071 (20 813 to 21 329)	497 (333 to 660)
Change in social welfare, $^a^	NA	NA	1899 (1682 to 2116)

Abbreviation: NA, not applicable.

^a^ Social welfare = $100 000 × (Life expectancy) – Cost.

To evaluate the potential implications for health disparities, we stratified the analysis by race and ethnicity. Among 513 Black patients in the testing cohort, 309 (60.2%) were found to have a higher-performing hospital. Simulated reassignment to an alternative hospital was associated with a 23.5% (95% CI, 19.3%-32.9%) relative reduction and 0.26% (95% CI, 0.21%-0.30%) absolute reduction in mortality risk for Black patients. Black patients experienced mean social welfare gains of $2427 (95% CI, $1697-$3158) compared with $1899 (95% CI, $1682-$2116) for White patients. For Black patients, selecting a higher-quality hospital was associated with a decreased mortality risk from 1.10% (95% CI, 0.68%-1.53%) at the chosen hospital to 0.84% (95% CI, 0.45%-1.23%; *P* < .001) at the highest-performing hospital but was not associated with increased costs ($349; 95% CI, –$183 to $882; *P* = .20). For White patients, selecting a higher-quality hospital was associated with a decreased mortality risk from 0.88% (95% CI, 0.78%-0.99%) at the chosen hospital to 0.65% (0.55%-0.74%) at the highest-performing hospital, while hospital costs were increased by $497 (95% CI, $333-$660) (Table 4).

## Discussion

In this study, referral of patients with colorectal cancer to the highest-performing local hospitals was associated with improved outcomes for both society (social welfare) and the patient (mortality) despite increased costs of care. We found an alternative higher-performing local hospital for over half of the study cohort, with the highest-performing hospital only a median (IQI) of 1.9 (0.5-3.5) miles farther than the original chosen hospital. Extrapolating the findings to the population of 5000 surgical patients with colorectal cancer who were treated in Florida over a single year showed that the use of optimal hospital selection for every patient could result in a societal savings of approximately $9 765 000 (95% CI, $8 720 000-$10 810 000).

Patients eligible for movement to a higher-performing hospital were older, had more comorbidities, and more often had Medicare coverage than patients who were already receiving treatment at their highest-performing local hospital. These findings are consistent with those of prior research that found that older patients were more likely than younger patients to receive care at a hospital closest to them.^40^ Older adults may experience difficulty navigating publicly reported hospital quality information, relying instead on proximity or word-of-mouth to select a hospital.^9^ However, even the recommendations of referring physicians may be informed more by reputation and subjective experiences than objective quality data.^13^

This study found that 60.2% of Black patients could receive care at a better local hospital. Although in-hospital mortality risk remained slightly higher for Black patients compared with White patients, optimal hospital selection resulted in similar absolute reductions in mortality for Black and White patients. Study findings were consistent with reports in prior research that care at lower-quality hospitals contributed to disparities in both short-term^41,42^ and long-term survival^43^ after surgery. We extended these findings by quantifying the potential benefits and costs of using outcomes data to facilitate choice of hospitals for Black patients with colorectal cancer. We also found that optimal hospital selection may be necessary but insufficient on its own in achieving health equity. Given that differences in quality among multiple phases of surgical care are factors in disparities, additional efforts addressing the disparities in surgical referrals^44^ and preoperative medical optimization are necessary.

Prior studies have also found improved outcomes by optimizing hospital selection for oncologic surgery. Simulated choice of higher-quality hospitals that was based on risk-standardized mortality ratios^45^ and Centers for Medicare and Medicaid Services star ratings^46^ showed improved surgical outcomes for patients with colorectal cancer. While these 2 studies showed the generalized implications of care at higher-performing hospitals, neither study considered the cost or geographic location and, therefore, may have reallocated a patient to a hospital that may not be practical or even feasible. The present study confirmed the presence of meaningful variation in hospital quality even within each patient’s geographic area and described alternative hospital selection strategies that considered convenience for the patients.

Among the hospitals included in this analysis, we found no single trait that distinguished high-performing from low-performing hospitals. Many hospitals regardless of bed size or cancer center status performed high-quality colorectal surgery. These findings highlighted the utility of procedure-specific performance metrics for individual hospitals. Although hospital capacity may constrain the ability of higher-performing hospitals to accept additional referrals, this study showed that a median of 13 additional patients annually would be referred to the highest-performing hospitals, which seemed unlikely to overwhelm a single hospital.

Interactions between patient and clinician factors may also play a role in hospital selection and a hospital’s ability to provide high-quality care that was tailored to the individual patient. The study simulated hospital choice using only risk-adjusted outcomes data and geographic location. While, in general, most patients are motivated to seek care at the highest-quality hospitals,^10^ patients consider many other factors when selecting a hospital, such as familiarity with a health system, financial limitations, or travel constraints. While insurance networks may limit hospital choice, most private insurance plans cover the majority of hospitals within their ratings area.^47^ Thus, publicly reported information on hospital performance for individual procedures may enhance a patient’s ability to weigh their potential outcomes against other considerations when choosing a hospital. As simulated in this study, incorporating quality information into the hospital selection process had an association with improved outcomes for patients and society.

### Limitations

This study has several limitations. First, we included patients from a single state, which may not generalize to other regions or rural populations with fewer local hospitals. Additionally, the cohort was limited to patients who underwent elective operations and may not apply to patients who present acutely and may be at higher risk of postoperative complications. Second, the data lacked detailed oncologic staging, which would have facilitated life expectancy estimation. However, the results were robust to alternative specifications of life expectancy in sensitivity analyses. Third, given that we included only patients who underwent surgical resection, the study did not address possible racial disparities in surgical referral or treatment use. Fourth, we likely underestimated the true benefits of data-driven hospital selection by studying only in-hospital mortality during the index admission for surgery.^47^ Research examining postoperative complications, readmissions, recurrence, or long-term survival is needed to show the long-term implications of data-driven hospital selection.

## Conclusions

In this economic evaluation, we found that procedure-specific hospital performance as the primary factor in the choice of a local hospital for colorectal cancer surgery was associated with improved outcomes for both patients and society. The study supported the use of outcomes data by patients and referring physicians to make an informed decision when selecting a hospital for care. If the benefits simulated in this study translated to improved outcomes in practice, this approach to hospital choice could be used to transform care and guide policy in colorectal cancer surgery.

## Notes

Supplement 1. **eFigure.** Simulation Analysis Methods **eTable 1.** ICD-10 Inclusion Codes for Colorectal Resection **eTable 2.** ICD-10 Diagnosis Codes to Identify Patients With Neoplasm of the Colon or Rectum **eTable 3.** Population Characteristics Stratified by Race **eTable 4.** Characteristics of Hospitals That Gained or Lost Patients Following Optimized Hospital Selection **eTable 5.** Change in Social Welfare With Optimized Hospital Selection Across the Population for Different Valuations of One Life Year **eTable 6.** Sensitivity Analysis Where Life Expectancy Estimations for Patients With Benign or In Situ Neoplasms Are Not Adjusted for Colorectal Cancer Severity

Supplement 2.
Data Sharing Statement
