# Supplementary material for: Comparison of Simulated Outcomes of Colorectal Cancer Surgery at the Highest-Performing vs Chosen Local Hospitals
Source: JAMA Netw Open. 2023 Feb 15;6(2):e2255999. doi: 10.1001/jamanetworkopen.2022.55999 (PMC9932827; doi:10.1001/jamanetworkopen.2022.55999)
Supplement: Supplement 1. — eFigure. Simulation Analysis Methods eTable 1. ICD-10 Inclusion Codes for Colorectal Resection eTable 2. ICD-10 Diagnosis Codes to Identify Patients With Neoplasm of the Colon or Rectum eTable 3. Population Characteristics Stratified by Race eTable 4. Characteristics of Hospitals That Gained or Lost Patients Following Optimized Hospital Selection eTable 5. Change in Social Welfare With Optimized Hospital Selection Across the Population for Different Valuations of One Life Year eTable 6. Sensitivity Analysis Where Life Expectancy Estimations for Patients With Benign or In Situ Neoplasms Are Not Adjusted for Colorectal Cancer Severity [file jamanetwopen-e2255999-s001.pdf]

# Supplemental Online Content

Finn CB, Wirtalla C, Roberts SE, et al. Comparison of simulated outcomes of colorectal cancer surgery at the highest-performing vs chosen local hospitals. *JAMA Netw Open*. 2023;6(2):e2255999. doi:10.1001/jamanetworkopen.2022.55999

**eFigure.** Simulation Analysis Methods

**eTable 1.** ICD-10 Inclusion Codes for Colorectal Resection

**eTable 2.** ICD-10 Diagnosis Codes to Identify Patients With Neoplasm of the Colon or Rectum

**eTable 3.** Population Characteristics Stratified by Race

**eTable 4.** Characteristics of Hospitals That Gained or Lost Patients Following Optimized Hospital Selection

**eTable 5.** Change in Social Welfare With Optimized Hospital Selection Across the Population for Different Valuations of One Life Year

**eTable 6.** Sensitivity Analysis Where Life Expectancy Estimations for Patients With Benign or In Situ Neoplasms Are Not Adjusted for Colorectal Cancer Severity

This supplementary material has been provided by the authors to give readers additional information about their work.

© 2023 Finn CB et al. *JAMA Network Open.*

**eFigure: Simulation analysis methods.**

Hierarchical logistic regression analysis was used to decompose the risk of an adverse event into the patient's fixed effect and hospital random effect. Sampling from the hospital random effect distribution provided random effect estimates that were used to simulate hospital performance in the testing cohort. Combining the fixed effects of the testing cohort patients with the random effect distribution provided the posterior probability of an adverse event. A Bernoulli trial (e.g., coin flip) was performed using the posterior probabilities to obtain each patient's average outcome at each potential hospital. RE: Random effect. FE: Fixed effect.

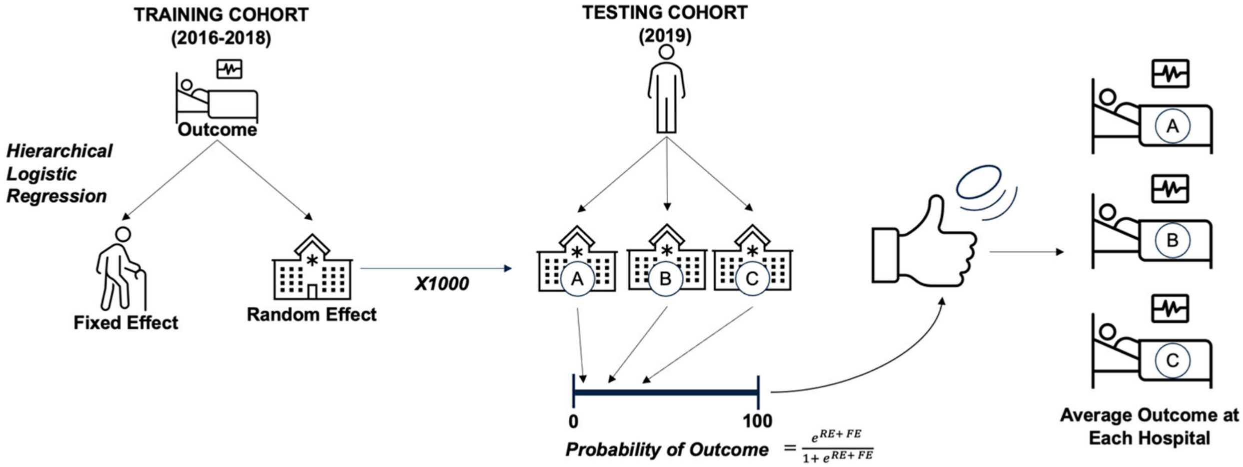


© 2023 Finn CB et al. *JAMA Network Open.*

**eTable 1:** ICD-10 inclusion codes for colorectal resection.

| ICD-10 Code | Description |
|---|---|
| **Colectomy** | |
| 0DBE0ZX | Excision of Large Intestine, Open Approach, Diagnostic |
| 0DBE0ZZ | Excision of Large Intestine, Open Approach |
| 0DBE4ZX | Excision of Large Intestine, Percutaneous Endoscopic Approach, Diagnostic |
| 0DBE4ZZ | Excision of Large Intestine, Percutaneous Endoscopic Approach |
| 0DBE7ZZ | Excision of Large Intestine, Via Opening |
| 0DBF0ZX | Excision of Right Large Intestine, Open Approach, Diagnostic |
| 0DBF0ZZ | Excision of Right Large Intestine, Open Approach |
| 0DBF4ZX | Excision of Right Large Intestine, Percutaneous Endoscopic Approach, Diagnostic |
| 0DBF4ZZ | Excision of Right Large Intestine, Percutaneous Endoscopic Approach |
| 0DBF7ZZ | Excision of Right Large Intestine, Via Opening |
| 0DBG0ZX | Excision of Left Large Intestine, Open Approach, Diagnostic |
| 0DBG0ZZ | Excision of Left Large Intestine, Open Approach |
| 0DBG4ZX | Excision of Left Large Intestine, Percutaneous Endoscopic Approach, Diagnostic |
| 0DBG4ZZ | Excision of Left Large Intestine, Percutaneous Endoscopic Approach |
| 0DBG7ZZ | Excision of Left Large Intestine, Via Opening |
| 0DBH0ZX | Excision of Cecum, Open Approach, Diagnostic |
| 0DBH0ZZ | Excision of Cecum, Open Approach |
| 0DBH4ZX | Excision of Cecum, Percutaneous Endoscopic Approach, Diagnostic |
| 0DBH4ZZ | Excision of Cecum, Percutaneous Endoscopic Approach |
| 0DBH7ZZ | Excision of Cecum, Via Natural or Artificial Opening |
| 0DBK0ZZ | Excision of Ascending Colon, Open Approach |
| 0DBK4ZZ | Excision of Ascending Colon, Percutaneous Endoscopic Approach |
| 0DBK7ZZ | Excision of Ascending Colon, Via Opening |
| 0DBL0ZX | Excision of Transverse Colon, Open Approach, Diagnostic |
| 0DBL0ZZ | Excision of Transverse Colon, Open Approach |
| 0DBL4ZX | Excision of Transverse Colon, Percutaneous Endoscopic Approach, Diagnostic |
| 0DBL4ZZ | Excision of Transverse Colon, Percutaneous Endoscopic Approach |
| 0DBL7ZZ | Excision of Transverse Colon, Via Opening |
| 0DBM0ZX | Excision of Descending Colon, Open Approach, Diagnostic |
| 0DBM0ZZ | Excision of Descending Colon, Open Approach |
| 0DBM4ZX | Excision of Descending Colon, Percutaneous Endoscopic Approach, Diagnostic |
| 0DBM4ZZ | Excision of Descending Colon, Percutaneous Endoscopic Approach |
| 0DBM7ZZ | Excision of Descending Colon, Via Opening |
| 0DBN0ZX | Excision of Sigmoid Colon, Open Approach, Diagnostic |
| 0DBN0ZZ | Excision of Sigmoid Colon, Open Approach |
| 0DBN4ZX | Excision of Sigmoid Colon, Percutaneous Endoscopic Approach, Diagnostic |
| 0DBN4ZZ | Excision of Sigmoid Colon, Percutaneous Endoscopic Approach |

© 2023 Finn CB et al. *JAMA Network Open.*

| | |
|---|---|
| 0DBN7ZZ | Excision of Sigmoid Colon, Via Natural or Artificial Opening |
| 0DTE0ZZ | Resection of Large Intestine, Open Approach |
| 0DTE4ZZ | Resection of Large Intestine, Percutaneous Endoscopic Approach |
| 0DTE7ZZ | Resection of Large Intestine, Via Opening |
| 0DTF0ZZ | Resection of Right Large Intestine, Open Approach |
| 0DTF4ZZ | Resection of Right Large Intestine, Percutaneous Endoscopic Approach |
| 0DTF7ZZ | Resection of Right Large Intestine, Via Opening |
| 0DTG0ZZ | Resection of Left Large Intestine, Open Approach |
| 0DTG4ZZ | Resection of Left Large Intestine, Percutaneous Endoscopic Approach |
| 0DTG7ZZ | Resection of Left Large Intestine, Via Opening |
| 0DTH0ZZ | Resection of Cecum, Open Approach |
| 0DTH4ZZ | Resection of Cecum, Percutaneous Endoscopic Approach |
| 0DTH7ZZ | Resection of Cecum, Via Natural or Artificial Opening |
| 0DTK0ZZ | Resection of Ascending Colon, Open Approach |
| 0DTK4ZZ | Resection of Ascending Colon, Percutaneous Endoscopic Approach |
| 0DTK7ZZ | Resection of Ascending Colon, Via Opening |
| 0DTL0ZZ | Resection of Transverse Colon, Open Approach |
| 0DTL4ZZ | Resection of Transverse Colon, Percutaneous Endoscopic Approach |
| 0DTL7ZZ | Resection of Transverse Colon, Via Opening |
| 0DTM0ZZ | Resection of Descending Colon, Open Approach |
| 0DTM4ZZ | Resection of Descending Colon, Percutaneous Endoscopic Approach |
| 0DTM7ZZ | Resection of Descending Colon, Via Opening |
| 0DTN0ZZ | Resection of Sigmoid Colon, Open Approach |
| 0DTN4ZZ | Resection of Sigmoid Colon, Percutaneous Endoscopic Approach |
| 0DTN7ZZ | Resection of Sigmoid Colon, Via Opening |
| | |
| **Proctectomy** | |
| 0D1A4ZQ | Bypass Jejunum to Anus, Percutaneous Endoscopic Approach |
| 0D1B0ZQ | Bypass Ileum to Anus, Open Approach |
| 0D1B4ZQ | Bypass Ileum to Anus, Percutaneous Endoscopic Approach |
| 0D1A0ZQ | Bypass Jejunum to Anus, Open Approach |
| 0DTP0ZZ | Resection of Rectum, Open Approach |
| 0DTP4ZZ | Resection of Rectum, Percutaneous Endoscopic Approach |
| 0DTP7ZZ | Resection of Rectum, Via Natural or Artificial Opening |
| 0DTQ7ZZ | Resection of Anus, Via Natural or Artificial Opening |
| | |
| **Other Large Bowel** | |
| 0D1H0ZH | Bypass Cecum to Cecum, Open Approach |
| 0D1H0ZK | Bypass Cecum to Ascending Colon, Open Approach |
| 0D1H0ZL | Bypass Cecum to Transverse Colon, Open Approach |
| 0D1H0ZM | Bypass Cecum to Descending Colon, Open Approach |

© 2023 Finn CB et al. *JAMA Network Open.*

| | |
|---|---|
| 0D1H0ZN | Bypass Cecum to Sigmoid Colon, Open Approach |
| 0D1H0ZP | Bypass Cecum to Rectum, Open Approach |
| 0D1H4ZH | Bypass Cecum to Cecum, Percutaneous Endoscopic Approach |
| 0D1H4ZK | Bypass Cecum to Ascending Colon, Percutaneous Endoscopic Approach |
| 0D1H4ZL | Bypass Cecum to Transverse Colon, Percutaneous Endoscopic Approach |
| 0D1H4ZM | Bypass Cecum to Descending Colon, Percutaneous Endoscopic Approach |
| 0D1H4ZN | Bypass Cecum to Sigmoid Colon, Percutaneous Endoscopic Approach |
| 0D1H4ZP | Bypass Cecum to Rectum, Percutaneous Endoscopic Approach |
| 0D1K0ZK | Bypass Ascending Colon to Ascending Colon, Open Approach |
| 0D1K0ZL | Bypass Ascending Colon to Transverse Colon, Open Approach |
| 0D1K0ZM | Bypass Ascending Colon to Descending Colon, Open Approach |
| 0D1K0ZN | Bypass Ascending Colon to Sigmoid Colon, Open Approach |
| 0D1K0ZP | Bypass Ascending Colon to Rectum, Open Approach |
| 0D1K4ZK | Bypass Ascending Colon to Ascending Colon, Percutaneous Endoscopic Approach |
| 0D1K4ZL | Bypass Ascending Colon to Trans Colon, Percutaneous Endoscopic Approach |
| 0D1K4ZM | Bypass Ascending Colon to Desc Colon, Percutaneous Endoscopic Approach |
| 0D1K4ZN | Bypass Ascending Colon to Sigmoid Colon, Percutaneous Endoscopic Approach |
| 0D1K4ZP | Bypass Ascending Colon to Rectum, Percutaneous Endoscopic Approach |
| 0D1L0ZL | Bypass Transverse Colon to Transverse Colon, Open Approach |
| 0D1L0ZM | Bypass Transverse Colon to Descending Colon, Open Approach |
| 0D1L0ZN | Bypass Transverse Colon to Sigmoid Colon, Open Approach |
| 0D1L0ZP | Bypass Transverse Colon to Rectum, Open Approach |
| 0D1L4ZL | Bypass Transverse Colon to Trans Colon, Percutaneous Endoscopic Approach |
| 0D1L4ZM | Bypass Transverse Colon to Desc Colon, Percutaneous Endoscopic Approach |
| 0D1L4ZN | Bypass Transverse Colon to Sigmoid Colon, Percutaneous Endoscopic Approach |
| 0D1L4ZP | Bypass Transverse Colon to Rectum, Percutaneous Endoscopic Approach |
| 0D1M0ZM | Bypass Descending Colon to Descending Colon, Open Approach |
| 0D1M0ZN | Bypass Descending Colon to Sigmoid Colon, Open Approach |
| 0D1M0ZP | Bypass Descending Colon to Rectum, Open Approach |
| 0D1M4ZM | Bypass Descending Colon to Desc Colon, Percutaneous Endoscopic Approach |
| 0D1M4ZN | Bypass Descending Colon to Sigmoid Colon, Percutaneous Endoscopic Approach |
| 0D1M4ZP | Bypass Descending Colon to Rectum, Percutaneous Endoscopic Approach |
| 0D1N0ZN | Bypass Sigmoid Colon to Sigmoid Colon, Open Approach |
| 0D1N0ZP | Bypass Sigmoid Colon to Rectum, Open Approach |
| 0D1N4ZN | Bypass Sigmoid Colon to Sigmoid Colon, Percutaneous Endoscopic Approach |
| 0D1N4ZP | Bypass Sigmoid Colon to Rectum, Percutaneous Endoscopic Approach |
| 0DME0ZZ | Reattachment of Large Intestine, Open Approach |
| 0DME4ZZ | Reattachment of Large Intestine, Percutaneous Endoscopic Approach |
| 0DMF0ZZ | Reattachment of Right Large Intestine, Open Approach |
| 0DMF4ZZ | Reattachment of Right Large Intestine, Percutaneous Endoscopic Approach |
| 0DMG0ZZ | Reattachment of Left Large Intestine, Open Approach |

© 2023 Finn CB et al. *JAMA Network Open.*

| | |
|---|---|
| 0DMG4ZZ | Reattachment of Left Large Intestine, Percutaneous Endoscopic Approach |
| 0DMH0ZZ | Reattachment of Cecum, Open Approach |
| 0DMH4ZZ | Reattachment of Cecum, Percutaneous Endoscopic Approach |
| 0DMK0ZZ | Reattachment of Ascending Colon, Open Approach |
| 0DMK4ZZ | Reattachment of Ascending Colon, Percutaneous Endoscopic Approach |
| 0DML0ZZ | Reattachment of Transverse Colon, Open Approach |
| 0DML4ZZ | Reattachment of Transverse Colon, Percutaneous Endoscopic Approach |
| 0DMM0ZZ | Reattachment of Descending Colon, Open Approach |
| 0DMM4ZZ | Reattachment of Descending Colon, Percutaneous Endoscopic Approach |
| 0DMN0ZZ | Reattachment of Sigmoid Colon, Open Approach |
| 0DMN4ZZ | Reattachment of Sigmoid Colon, Percutaneous Endoscopic Approach |
| 0DMP4ZZ | Reattachment of Rectum, Percutaneous Endoscopic Approach |
| 0DWE07Z | Revision of Autol Sub in Lg Intestine, Open Approach |
| 0DWE47Z | Revision of Autol Sub in Lg Intestine, Percutaneous Endoscopic Approach |
| | |
| **Other Small Bowel** | |
| 0D190Z9 | Bypass Duodenum to Duodenum, Open Approach |
| 0D190ZL | Bypass Duodenum to Transverse Colon, Open Approach |
| 0D194Z9 | Bypass Duodenum to Duodenum, Percutaneous Endoscopic Approach |
| 0D194ZL | Bypass Duodenum to Transverse Colon, Percutaneous Endoscopic Approach |
| 0D1A0ZB | Bypass Jejunum to Ileum, Open Approach |
| 0D1A0ZH | Bypass Jejunum to Cecum, Open Approach |
| 0D1A0ZK | Bypass Jejunum to Ascending Colon, Open Approach |
| 0D1A0ZL | Bypass Jejunum to Transverse Colon, Open Approach |
| 0D1A0ZM | Bypass Jejunum to Descending Colon, Open Approach |
| 0D1A0ZN | Bypass Jejunum to Sigmoid Colon, Open Approach |
| 0D1A0ZP | Bypass Jejunum to Rectum, Open Approach |
| 0D1A4ZB | Bypass Jejunum to Ileum, Percutaneous Endoscopic Approach |
| 0D1A4ZH | Bypass Jejunum to Cecum, Percutaneous Endoscopic Approach |
| 0D1A4ZK | Bypass Jejunum to Ascending Colon, Percutaneous Endoscopic Approach |
| 0D1A4ZL | Bypass Jejunum to Transverse Colon, Percutaneous Endoscopic Approach |
| 0D1A4ZM | Bypass Jejunum to Descending Colon, Percutaneous Endoscopic Approach |
| 0D1A4ZN | Bypass Jejunum to Sigmoid Colon, Percutaneous Endoscopic Approach |
| 0D1B0ZB | Bypass Ileum to Ileum, Open Approach |
| 0D1B0ZH | Bypass Ileum to Cecum, Open Approach |
| 0D1B0ZK | Bypass Ileum to Ascending Colon, Open Approach |
| 0D1B0ZL | Bypass Ileum to Transverse Colon, Open Approach |
| 0D1B0ZM | Bypass Ileum to Descending Colon, Open Approach |
| 0D1B0ZN | Bypass Ileum to Sigmoid Colon, Open Approach |
| 0D1B0ZP | Bypass Ileum to Rectum, Open Approach |
| 0D1B4ZB | Bypass Ileum to Ileum, Percutaneous Endoscopic Approach |

© 2023 Finn CB et al. *JAMA Network Open.*

| | |
|---|---|
| 0D1B4ZH | Bypass Ileum to Cecum, Percutaneous Endoscopic Approach |
| 0D1B4ZK | Bypass Ileum to Ascending Colon, Percutaneous Endoscopic Approach |
| 0D1B4ZL | Bypass Ileum to Transverse Colon, Percutaneous Endoscopic Approach |
| 0D1B4ZM | Bypass Ileum to Descending Colon, Percutaneous Endoscopic Approach |
| 0D1B4ZN | Bypass Ileum to Sigmoid Colon, Percutaneous Endoscopic Approach |
| 0D1B4ZP | Bypass Ileum to Rectum, Percutaneous Endoscopic Approach |
| 0DM80ZZ | Reattachment of Small Intestine, Open Approach |
| 0DM84ZZ | Reattachment of Small Intestine, Percutaneous Endoscopic Approach |
| 0DM90ZZ | Reattachment of Duodenum, Open Approach |
| 0DM94ZZ | Reattachment of Duodenum, Percutaneous Endoscopic Approach |
| 0DMA0ZZ | Reattachment of Jejunum, Open Approach |
| 0DMA4ZZ | Reattachment of Jejunum, Percutaneous Endoscopic Approach |
| 0DMB0ZZ | Reattachment of Ileum, Open Approach |
| 0DMB4ZZ | Reattachment of Ileum, Percutaneous Endoscopic Approach |
| 0DQC0ZZ | Repair Ileocecal Valve, Open Approach |
| 0DQC4ZZ | Repair Ileocecal Valve, Percutaneous Endoscopic Approach |
| 0DV80CZ | Restriction of Small Intestine with Extraluminal Device, Open Approach |
| 0DV80ZZ | Restriction of Small Intestine, Open Approach |
| 0DV84CZ | Restrict Small Intestine Extraluminal Device, Percutaneous Endoscopic |
| 0DV84ZZ | Restriction of Small Intestine, Percutaneous Endoscopic Approach |
| 0DV90CZ | Restriction of Duodenum with Extraluminal Device, Open Approach |
| 0DV90ZZ | Restriction of Duodenum, Open Approach |
| 0DV94CZ | Restrict of Duodenum with Extraluminal Device, Percutaneous Endoscopic Approach |
| 0DV94ZZ | Restriction of Duodenum, Percutaneous Endoscopic Approach |
| 0DVA0CZ | Restriction of Jejunum with Extraluminal Device, Open Approach |
| 0DVA0ZZ | Restriction of Jejunum, Open Approach |
| 0DVA4CZ | Restriction of Jejunum with Extraluminal Device, Percutaneous Endoscopic Approach |
| 0DVA4ZZ | Restriction of Jejunum, Percutaneous Endoscopic Approach |
| 0DVB0CZ | Restriction of Ileum with Extraluminal Device, Open Approach |
| 0DVB0ZZ | Restriction of Ileum, Open Approach |
| 0DVB4CZ | Restriction of Ileum with Extraluminal Device, Percutaneous Endoscopic Approach |
| 0DVB4ZZ | Restriction of Ileum, Percutaneous Endoscopic Approach |
| 0DVC0CZ | Restrict of Ileocecal Valve with Extraluminal Device, Open Approach |
| 0DVC0ZZ | Restriction of Ileocecal Valve, Open Approach |
| 0DVC4CZ | Restrict Ileocecal Valve w Extraluminal Device, Percutaneous Endoscopic |
| 0DVC4ZZ | Restriction of Ileocecal Valve, Percutaneous Endoscopic Approach |
| 0DW807Z | Revision of Autol Sub in Small Intestine, Open Approach |
| 0DW847Z | Revision of Autol Sub in Small Intestine, Percutaneous Endoscopic Approach |

© 2023 Finn CB et al. *JAMA Network Open.*

**eTable 2:** ICD-10 diagnosis codes to identify patients with neoplasm of the colon or rectum.

| Code | Description |
|---|---|
| **"Malignant neoplasm"** | |
| C180 | Malignant neoplasm of cecum |
| C181 | Malignant neoplasm of appendix |
| C182 | Malignant neoplasm of ascending colon |
| C183 | Malignant neoplasm of hepatic flexure |
| C184 | Malignant neoplasm of transverse colon |
| C185 | Malignant neoplasm of splenic flexure |
| C186 | Malignant neoplasm of descending colon |
| C187 | Malignant neoplasm of sigmoid colon |
| C188 | Malignant neoplasm of overlapping sites of colon |
| C189 | Malignant neoplasm of colon, unspecified |
| C19 | Malignant neoplasm of rectosigmoid junction |
| C20 | Malignant neoplasm of rectum |
| C210 | Malignant neoplasm of anus, unspecified |
| C211 | Malignant neoplasm of anal canal |
| C218 | Malignant neoplasm of overlapping sites of rectum, anus and anal canal |
| | |
| **"In situ neoplasms"** | |
| D01.0 | Carcinoma in situ of colon |
| D01.1 | Carcinoma in situ of rectosigmoid junction |
| D01.2 | Carcinoma in situ of rectum |
| D01.3 | Carcinoma in situ of anus and anal canal |
| D01.40 | Carcinoma in situ of unspecified part of intestine |
| | |
| **"Benign neoplasms"** | |
| D12 | Benign neoplasm of colon, rectum, anus and anal canal |
| D12.0 | Benign neoplasm of cecum |
| D12.1 | Benign neoplasm of appendix |
| D12.2 | Benign neoplasm of ascending colon |
| D12.3 | Benign neoplasm of transverse colon |
| D12.4 | Benign neoplasm of descending colon |
| D12.5 | Benign neoplasm of sigmoid colon |
| D12.6 | Benign neoplasm of colon, unspecified |
| D12.7 | Benign neoplasm of rectosigmoid junction |
| D12.8 | Benign neoplasm of rectum |
| D12.9 | Benign neoplasm of anus and anal canal |
| | |
| **"Neoplasm of uncertain behavior"** | |

© 2023 Finn CB et al. *JAMA Network Open.*

| | |
|---|---|
| D37.3 | Neoplasm of uncertain behavior of appendix |
| D37.4 | Neoplasm of uncertain behavior of colon |
| D37.5 | Neoplasm of uncertain behavior of rectum |
| D37.9 | Neoplasm of uncertain behavior of digestive organ, unspecified |
| | |
| **"Benign neuroendocrine tumor"** | |
| D3A.02 | Benign carcinoid tumors of the appendix, large intestine, and rectum |
| D3A.020 | Benign carcinoid tumor of the appendix |
| D3A.021 | Benign carcinoid tumor of the cecum |
| D3A.022 | Benign carcinoid tumor of the ascending colon |
| D3A.023 | Benign carcinoid tumor of the transverse colon |
| D3A.024 | Benign carcinoid tumor of the descending colon |
| D3A.025 | Benign carcinoid tumor of the sigmoid colon |
| D3A.026 | Benign carcinoid tumor of the rectum |
| D3A.029 | Benign carcinoid tumor of the large intestine, unspecified portion |
| | |
| **"Malignant neuroendocrine tumor"** | |
| C7A.02 | Malignant carcinoid tumors of the appendix, large intestine, and rectum |
| C7A.020 | Malignant carcinoid tumor of the appendix |
| C7A.021 | Malignant carcinoid tumor of the cecum |
| C7A.022 | Malignant carcinoid tumor of the ascending colon |
| C7A.023 | Malignant carcinoid tumor of the transverse colon |
| C7A.024 | Malignant carcinoid tumor of the descending colon |
| C7A.025 | Malignant carcinoid tumor of the sigmoid colon |
| C7A.026 | Malignant carcinoid tumor of the rectum |
| C7A.029 | Malignant carcinoid tumor of the large intestine, unspecified portion |
| | |
| **"Neoplasm of unspecified behavior"** | |
| D49.0 | Neoplasm of unspecified behavior of digestive system |
| | |
| **"Polyp"** | |
| K62.0 | Anal polyp |
| K62.1 | Rectal polyp |
| K63.5 | Polyp of colon |
| | |
| **"Secondary malignant neoplasm"** | |
| C78.5 | Secondary malignant neoplasm of large intestine and rectum |

© 2023 Finn CB et al. *JAMA Network Open.*

**eTable 3:** Population characteristics stratified by race.

| Characteristic | Black | White | Total | P-value |
|---|---|---|---|---|
| Number (%) | 2,232 (10.6) | 18,866 (89.4) | 21,098 (100.0) | |
| Age, mean (SD) | 63.8 (11.7) | 67.7 (11.9) | 67.3 (12.0) | <0.001 |
| Sex | | | | |
| Male | 1,039 (46.6) | 9,743 (51.6) | 10,782 (51.1) | <0.001 |
| Female | 1,193 (53.4) | 9,123 (48.4) | 10,316 (48.9) | |
| Principal payer | | | | |
| Medicare | 1,198 (53.7) | 11,956 (63.4) | 13,154 (62.3) | <0.001 |
| Medicaid | 144 (6.5) | 542 (2.9) | 686 (3.3) | |
| Commercial | 774 (34.7) | 5,807 (30.8) | 6,581 (31.2) | |
| Self | 29 (1.3) | 144 (0.8) | 173 (0.8) | |
| Other | 87 (3.9) | 417 (2.2) | 504 (2.4) | |
| Elixhauser comorbidities | | | | |
| 0 | 92 (4.1) | 1,011 (5.4) | 1,103 (5.2) | <0.001 |
| 1 | 302 (13.5) | 3,211 (17.0) | 3,513 (16.7) | |
| 2 | 470 (21.1) | 4,224 (22.4) | 4,694 (22.2) | |
| 3+ | 1,368 (61.3) | 10,420 (55.2) | 11,788 (55.9) | |
| Frailty | 56 (2.5) | 337 (1.8) | 393 (1.9) | 0.02 |
| Tumor Type | | | | |
| Malignant neoplasm of colon | 1,281 (57.4) | 9,960 (52.8) | 11,241 (53.3) | <0.001 |
| Malignant neoplasm of rectosigmoid junction | 124 (5.6) | 1,289 (6.8) | 1,413 (6.7) | |
| Malignant neoplasm of rectum | 184 (8.2) | 2,187 (11.6) | 2,371 (11.2) | |
| Benign or in situ neoplasm | 643 (28.8) | 5,430 (28.8) | 6,073 (28.8) | |
| Metastatic Cancer | 361 (16.2) | 2,597 (13.8) | 2,958 (14.0) | 0.002 |
| Procedure Type | | | | |
| Colectomy | 2,091 (93.7) | 17,308 (91.7) | 19,399 (91.9) | 0.006 |
| Other Large Bowel | < 11 (.) | 67 (0.4) | <78 (.) | |
| Proctectomy | 128 (5.7) | 1,443 (7.6) | 1,571 (7.4) | |
| Other Small Bowel | < 11 (.) | 48 (0.3) | < 59 (.) | |
| Laparoscopic | 1,119 (50.1) | 10,119 (53.6) | 11,238 (53.3) | 0.002 |

Data presented as number (percentage) unless otherwise specified. SD: Standard deviation. IQI: inter-quartile interval.

© 2023 Finn CB et al. *JAMA Network Open.*

**eTable 4:** Characteristics of hospitals that gained or lost patients following optimized hospital selection.

| | **Net negative or no change** | **Net Positive** | **P-Value** |
|---|---|---|---|
| Number (%) | 72 (40.4) | 106 (59.6) | |
| Location Type | | | 0.41 |
| Urban | 72 (100.0) | 105 (99.1) | |
| Rural | 0 (0.0) | < 10 (.) | |
| ACS Approved Cancer Program | 46 (63.9) | 35 (33.0) | <0.001 |
| Cancer Center Affiliate | 22 (30.6) | 62 (58.5) | <0.001 |
| Bed Size | | | <0.001 |
| Small (<100) | < 10 (.) | 22 (20.8) | |
| Medium (100-299) | 29 (40.3) | 52 (49.1) | |
| Large (300+) | 42 (58.3) | 32 (30.2) | |
| Patients moved during simulation, mean (SD) | -20.6 (22.6) | 14.0 (7.9) | <0.001 |
| Patients moved during simulation, median (IQI) | -13.0 (-27.0; -3.3) | 13.0 (7.0; 19.3) | <0.001 |

Data presented as number (percentage) unless otherwise specified. SD: Standard deviation. IQI: inter-quartile interval.

© 2023 Finn CB et al. *JAMA Network Open.*

**eTable 5:** Change in social welfare with optimized hospital selection across the population for different valuations of one life year.

| | **$50,000** | **$100,000** | **$150,000** |
|---|---|---|---|
| Total Population | 736 (565-906) | 1,953 (1,744-2,162) | 3,170 (2,909-3,432) |
| By Race | | | |
| Black | 1,039 (453-1625) | 2,427 (1,697-3,158) | 3,815 (2,892-4,739) |
| White | 701 (523-879) | 1,899 (1,682-2,116) | 3,097 (2,826-3,368) |

Data presented as estimate (95% confidence interval).

© 2023 Finn CB et al. *JAMA Network Open.*

**eTable 6:** Sensitivity analysis where life expectancy estimations for patients with benign or in situ neoplasms are not adjusted for colorectal cancer severity.

| | **$50,000** | **$100,000** | **$150,000** |
|---|---|---|---|
| Total Population | 818 (645-991) | 2,117 (1,900-2,333) | 3,416 (3,142-3,691) |
| By Race | | | |
| Black | 1,112 (523-1,700) | 2,572 (1,836-3,309) | 4,033 (3,098-4,967) |
| White | 784 (603-965) | 2,065 (1,839-2,291) | 3,346 (3,059-3,632) |

Data presented as estimate (95% confidence interval).

© 2023 Finn CB et al. *JAMA Network Open.*
