# Supplementary material for: Comparison of Simulated Outcomes of Colorectal Cancer Surgery at the Highest-Performing vs Chosen Local Hospitals
Source: JAMA Netw Open. 2023 Feb 15;6(2):e2255999. doi: 10.1001/jamanetworkopen.2022.55999 (PMC9932827; doi:10.1001/jamanetworkopen.2022.55999)
Supplement: Supplement 2. — Data Sharing Statement [file jamanetwopen-e2255999-s002.pdf]

Data Sharing Statement

Finn. Comparison of Simulated Outcomes of Colorectal Cancer Surgery at the Highest-Performing vs Chosen Local Hospitals. *JAMA Netw Open*. Published February 15, 2023. doi:10.1001/jamanetworkopen.2022.55999

Data
Data available: No

Additional Information
Explanation for why data not available: To maintain patient confidentiality and comply with the terms of our data use agreement, the data used in this study will not be publicly shared. The data used for this manuscript was derived from a limited data set supplied by the Florida Agency for Health Care Administration (AHCA).
